# Genotypic and phenotypic β-lactam resistance and presence of PVL gene in Staphylococci from dry bovine udder

**DOI:** 10.1371/journal.pone.0187277

**Published:** 2017-11-01

**Authors:** Vinodkumar Kulangara, Neetha Nair, Asok Sivasailam, Suchithra Sasidharan, Justin Davis Kollannur, Radhika Syam

**Affiliations:** Department of Veterinary Epidemiology and Preventive Medicine, College of Veterinary and Animal Sciences, Mannuthy, Thrissur, Kerala, India; Universita degli Studi di Napoli Federico II, ITALY

## Abstract

Dairy cows affected with subclinical mastitis can be sources of virulent, antimicrobial-resistant Staphylococci to humans because of the excretion of the bacteria through their milk. This study focussed on the phenotypic and genotypic antibiotic resistance patterns of Staphylococci isolated from dairy cows in early dry period. Among 96 isolates of Gram positive cocci from 157 cows, 76 were identified as Coagulase Negative Staphylococci and the remaining 20 were *Staphylococcus aureus*. Typical amplicons of coagulase gene were obtained for all 20 samples of *S*. *aureus* with three major coagulase types being identified as giving 627 bp (40%), 910 bp (35%) and 710 bp (25%) long PCR products. The *groEL* gene was amplified in PCR of all 76 isolates of Coagulase Negative Staphylococci, and incubation of PCR products with restriction enzyme PvuII yielded three distinct PCR-RFLP fragment patterns bearing resemblance to *S*. *chromogenes* and *S*. *hyicus*. Highest sensitivity of Coagulase Negative Staphylococci was noted for Azithromycin (92.5%) and the least to Tetracyclines (76.3%), whereas for *S*. *aureus*, it was Cefoperazone (95%) and Azithromycin (72.2%) respectively. Phenotypic resistance to Oxacillin (25 isolates), and Cefoxitin (11 isolates) was detected by dilution method with a commercial strip (Ezy MIC^TM^). Genotypic resistance to β-Lactam antibiotics was found in 65 (34 with *mecA* gene and 31 with *blaZ* gene) isolates. Eighteen isolates possessed both the genes, with the PVL gene for virulence being detected in five of them. Nine isolates which had *mecA* gene were phenotypically susceptible to oxacillin while phenotypic resistance to oxacillin was observed in seven isolates that did not have either *mec*A or *blaZ* gene. This is the first report of persistent Staphylococcal infections possessing PVL gene and high level of genotypic resistance to β-Lactam antibiotics in small- holder dairy cattle from India.

## Introduction

Community Associated Methicillin Resistant Staphylococci (CA-MRSA) and Livestock Associated Methicillin Resistant Staphylococci (LA-MRSA) outbreaks with fatal nosocomial pneumonia, food-borne infections and post-surgical complications in human patients have been reported all over the world [1]. Infection by such antibiotic resistant Staphylococci pose a major threat to human health and dairy cows are recognised as one of the potential reservoirs [2, 3]. *Staphylococcus aureus* (SA) is a major pathogen causing subclinical mastitis in bovines, while Coagulase Negative Staphylococci (CNS) are considered minor pathogens causing increase in somatic cell count with wide species variations. Staphylococci invade and colonise the mammary gland of cows during lactation, persist in the udder parenchyma throughout the life of the animal, and are shed continuously through the milk [4]. Antibiotic-resistant strains, particularly those resistant to β-lactam group of antibiotics have emerged in Staphylococci possibly as a result of regular antimicrobial use in dairy farms to control them. Their presence in bovine milk and dairy environment poses a potential risk to consumers, farmers, farm workers, and veterinarians alike who are exposed to the affected cattle.

Recently, the Indian Network for Surveillance of Antimicrobial Resistance in India has stressed the need to study epidemiology of MRSA [5]. India is the largest producer and consumer of milk in the world, and the practice of consuming raw milk is still prevalent among many Indian communities. The potential significance of milk as a source of antibiotic resistant Staphylococci to humans has not still had the attention it warrants despite well-established risks associated with consumption of raw or unpasteurised milk. The incidence rate of CA MRSA varies from 25% in Western parts of India to 50% in South India in human patients [5] while no reports are available on LA MRSA.

The continuing emergence of newer genetic clusters in Staphylococci demands consideration of genotypic resistance together with molecular level profile to identify relevant, evolving genotypes. The coagulase gene is recognized as a suitable target for molecular level profiling of phenotypically identified *S*. *aureus* isolates [6]. PCR-RFLP of the *groEL* gene was found to be capable of identifying 100% of CNS species isolated from human clinical samples and from milk samples of dairy cows with intra-mammary infection, and hence is considered to be an ideal universal target for identification of CNS to the species level [7].

Resistance to penicillin as developed by staphylococcus is mediated by two *mec*hanisms: either by the secretion of an enzyme β-lactamases which inactivates the antibiotic by hydrolysis of its β-lactam ring, and is encoded by the gene *blaZ* in a plasmid or chromosome [8], or by the production of a penicillin binding protein (PBP2A) encoded by a gene *mec*A, embedded in a mobile genetic element on the bacterial chromosome called Staphylococcal Chromosome Cassette *mec* or SCC*mec* [6]. Isolates of Staphylococcus are characterized as Methicillin Resistant (MRSA for *S*. *aureus* and MRCNS for coagulase negative Staphylococci) if they show the presence of the *mecA* gene and display phenotypic resistance to oxacillin/methicillin. The TetK gene found in Staphylococci codes for the energy dependant efflux of tetracycline conferring resistance.

Existence of virulence factors in MRSA is another area of importance as pathogenicity of the bacteria depends on their production. Panton–Valentine leukocidin (PVL) is a *S*. *aureus*-specific, bi-component cytotoxin that causes leucocyte destruction and tissue necrosis [9] in human patients. Inhibition of immune response by PVL also aid in bacterial persistence and extension of inflammatory process in soft tissues, bone and joints. While the strains of community-associated MRSA, the majority of which carry genes encoding Panton–Valentine leucocidin, are spreading rapidly in human populations, only sporadic cases have been reported in animals to date [10].

High prevalence of Staphylococcal infections were found when secretions from a large number of dry cows maintained under small holder systems in an area of Kerala State, India were screened in this study. Phenotypic and genotypic antibiogram profile of the isolates revealed resistance to a number of commonly used antibiotics in human medicine.

## Materials and methods

### Animals

One hundred and fifty seven pregnant dairy cows within 5 days of cessation of their lactation were included in this study. Details of all the small holder dairy units (2–3 cows/holding) from an area of twenty Sq. km in the district of Thrissur in Kerala State, India, were collected and each unit was visited frequently. The animals belonged to the cross-bred category of non-descript breeds with either Jersey or Holstein–Friesian breeds. Udder secretions were collected from every one of the 157 pregnant cows within five days of cessation of their lactation. This study did not require any specific permissions since it involved only a non-invasive collection of samples from a domestic species. Administrative and technical sanction to the work was granted as per order KVASU/DAR/R2/116/2011 dated 01/09/11 of DAR, KVASU of the Director of Academics and Research, Kerala Veterinary and Animal Sciences University.

### Bacterial isolates

Mammary gland secretions of cows were collected in sterile vials and sealed aseptically. Bacterial cultures were obtained by inoculating the samples on Brain Heart Infusion Agar (Hi Media, India) and incubating for 24 h at 37°C. The plates were examined for growth and a cut-off value of 100CFU/ml of secretion was used. Pure cultures were maintained after re-inoculating individual colonies on Blood agar and Mannitol Salt agar. Selective isolation of *Staphylococcus* sp. was done on Mannitol salt agar and Baird-Parker medium containing one per cent Potassium tellurite. Further identification was carried out by Grams staining and biochemical tests for catalase, oxidase, oxidation- fermentation and production of acid from various sugars. Tube coagulase test was employed for initial discrimination of *S*. *aureus* and coagulase negative *Staphylococcus* isolates.

### Antimicrobial susceptibility of Staphylococci

The disc diffusion method [11] was employed for determining the antibiotic susceptibility. Discs containing Ampicillin (10 mcg), Penicillin G (10 units), Streptomycin (10 mcg), Tertracycline (30mcg), Azithromycin (30 mcg), Cefoperazone (5mcg) and Chloramphenicol (30 mcg) were used. Results were interpreted using the charts provided by the manufacturers of antibiotic discs (Hi Media, India).

### Phenotypic resistance to Oxacillin and Cefoxitin

Quantitative determination of susceptibility of bacteria to oxacillin and Cefoxitin was done using an Ezy MIC™ strip (Oxacillin Ezy MIC^TM^ Strip (OXA) and Cefoxitin Ezy MIC ^TM^ 0.016–256 mcg/ml strips, Hi Media, India) according to the manufacturer’s instructions. The strip comprised of a predefined quantitative gradient which was used to determine the Minimum Inhibitory Concentration (MIC) in mcg/ml of the two antimicrobial agents against Staphylococci. Mueller-Hinton Agar with 2% NaCl added was the media used and the tests were incubated at 35°C for 24 hours. Interpretation was done using the following interpretive criteria for susceptibility categorization as per Clinical and Laboratory Standards Institute (CLSI) guidelines, 2011 [12].

*S*. *aureus*—S <_ 2 R > _ 4

Coagulase-negative Staphylococci S <_ 0.25 R > _ 0.5

### Extraction of genomic DNA and PCR

Overnight cultures of samples were prepared in Brain Heart Infusion broth. DNA was isolated using the DNeasy blood and tissue DNA extraction kit (Qiagen) according to the manufacturer’s instructions. Lysis of the bacterial cell wall was enhanced by adding Lysostaphin (Sigma- Aldrich) to the lysis buffer at a concentration of 10μg/ml. The primers used and protocols followed for each PCR is given in Table 1. The restriction fragment length polymorphism of *groEL* Chaperonin gene in CNS was studied using PvuII restriction endonuclease (Fermentas, Germany), as per the prescribed protocol [6].

**Table 1:** Target genes and primers used to identify S. aureus and coagulase-negative Staphylococci by PCR.

Sl No	Target gene	Primer sequences (5’-3’)	Amplicon size (bp)	Reference for protocols
1	Coagulase	F- ATAGAGATGCTGGTACAGG R- GCTTCCGATTGTTCGATGC	627–910	[6]
2	*groEL*	F-AIIIIGCIGGIGA(TC)GGIACIACIAC R-(TC)(TG)I(TC)(TG)ITCICC(AG)AAI CCIGGIGC (TC)TT	550	[13]
3	*blaZ*	F-AAAATCGATGGTAAAGGTTGGC R-AGTTCTGCAGTACCGGA-TTTGC	639	[14]
4	*mec*A	F-GTAGCGACAATAGGTAATAGT R-GTAGTGACAATAAACCTCCTA	532	[15]
5	TetK	F-GCTGGACAAAACTTCTTGGAATAT R-GTAGTGACAATAAACCTCCTA	360	[16]
6	PVL	F-GCTGGACAAAACTTCTTGGAATAT R-GATAGGACACCAATAAATTCTGGATTG	433	[17]

## Results

### Bacterial isolates

Pure culture was yielded by 152 of 157 samples. Gram positive cocci were the most numerous (70.4%) followed by Gram negative isolates (13.8%). Among the Gram positive cocci, 96 (63.2%) belonged to Staphylococci, out of which *S*. *aureus* was recovered from 20 (20.8%) samples and 76 (79.2%) were coagulase negative.

### Antimicrobial susceptibility

Majority of isolates was sensitive to Cefoperazone (95%), followed by Streptomycin (94.4%), while Azithromycin (72%) was the least effective molecule against *S*. *aureus* according to the disc diffusion results. Among the CNS, the isolates were most susceptible to Azithromycin (92.5%) and highest % of antimicrobial resistance was found for Tetracycline (76.3%). Percentage susceptibility of isoltes is given in Table 2.

**Table 2:** Frequencies of antimicrobial resistance identified by disc diffusion of *S*. *aureus* and coagulase negative Staphylococci isolated from dry cows in India.

Antimicrobial Agent	Penicillin G	Streptomycin	Ampicillin	Tetracycline	Azithromycin	Cefoperazone	Chloramphenicol
SA	83.3	94.4	85	85	72.2	95	80
CNS	80.6	89.5	77.6	76.3	92.5	89.5	84.2

Resistance to oxacillin was found in 25 isolates by the gradient method, while 11 isolates were resistant to Cefoxitin, and three isolates were resistant to both. (S1 Fig).

### Molecular characterisation by PCR and PCR-RFLP

#### Coagulase typing

Typical amplicons of coagulase gene were obtained for all the 20 samples of *S*. *aureus* screened. However, the genetic diversity of the isolates was assessed using the size of the amplicon. The PCR product ranged between 600bp to 900bp for different strains of *S*. *aureus*. Three major coagulase types were identified. Eight (40%) isolates gave a 627bp long PCR product. Seven (35%) isolates revealed a 910bp amplicon while twenty five % of isolates carried a 710bp long coagulase gene (Table 3).

**Table 3:** Frequencies of different Staphylococci isolated from udder secretions of 157 dry cows in India.

Staphylococci 96 (63.1%)	*Staphylococcus aureus* 20 (20.8%)	Coagulase PCR product 627bp 8 (40%)
		Coagulase PCR product 710bp 5 (25%)
		Coagulase PCR product 910bp 7 (35%)
	Coagulase Negative Staphylococci 76 (79.2%)	*S*. *hyicus* 14 (18.4%)
		*S*. *chromogenes* 8 (10.5%)
		Unidentified 54 (71.1%)

#### RFLP of *groEL* gene

Single distinct bands of amplicons of the desired size (550bp) were obtained for all the 76 isolates of CNS (S2 Fig). The PCR-RFLP yielded 3 distinct patterns after partial digestion with restriction endonuclease Pvu II (S3 Fig). Thirty % of samples gave intact products after digestion. Fragments of around 400bp and 150 bp were obtained for 40% of the samples and rest yielded partially digested products of size 500bp, 400bp and 250bp (Table 3).

#### *BlaZ*, mecA and TetK gene based PCR

Amplification of *BlaZ* gene (639 bp) was evident in 18 isolates of *S*. *aureus* and 13 isolates of CNS (S4 Fig), while the 532 bp product for *mec*A gene was obtained in 19 isolates of *S*. *aureus* and 15 isolates of CNS (S5 Fig). Simultaneous amplification of both *mec*A and *BlaZ* genes were positive in 18 isolates. The gene TetK was not amplified in any sample.

#### PVL gene based PCR

Five isolates, all of them positive for both *mec*A and *BlaZ* gene, shown a product of the expected size of 433 bp, indicating the presence of PVL gene [17].

## Discussion

This is the first report of persistent infection of dry bovine udders by Staphylococci having genes for methicillin resistance and the virulence factor PVL. Routine prevalence studies based upon detection of Staphylococci in milk during lactation find it difficult to differentiate between incidental transient infection or contamination during milking procedures and the existing infections. This study avoids this error by taking only those organisms present during the dry period into consideration [18]. The percentage (27.9) of phenotypic resistance of Staphylococci to oxacillin detected in our study was similar to reports from Uruguay (22%) [19] and Argentina (27.6%) [20], but lower than for Korea (60.2%) [8]. Phenotypic resistance to Cefoxitin was 15.8%, indicating presence of *mec*A or *mec*C MRSA [21] while 3 isolates were resistant to both antibiotics.

Nine isolates which had a *mecA* gene were phenotypically susceptible to oxacillin. Similar findings have been reported in two isolates from human samples from a nearby referral hospital, and further investigation before initiating treatment of infections with such organisms was recommended [22]. Phenotypic resistance to oxacillin was observed in seven isolates that did not have either *mec*A or *blaZ* gene. The blaZ gene-negative, but oxacillin-resistant Staphylococcus isolates which had been reported earlier [6] were assumed to be β-lactamase hyper producing strains by the authors since all of them were susceptible to amoxicillin-clauvulanate combination. We did not use this particular antibiotic disc since our study concentrated on antibiotics which are available as intra-mammary preparations. Another observation by the same authors, that MRSA generally exhibit resistance to tetracyclines, also could not be confirmed by our findings since none of the isolates in our study had the tetK gene.

This is also the first report of PVL gene in *S*. *aureus* from dairy cattle in India. Presence of the PVL gene in methicillin succeptible [23] and methicillin resistant [24] *S*. *aureus* from other animals has been previously reported, while others [25] have found presence of PVL gene to be independent of specific genetic backgrounds like *mec*A gene.

Reported bacterial resistance levels to different antibiotics, as determined by disc diffusion method differ between studies [3, 14, 15, 26]. One reason for the comparatively low susceptibility of isolates in this study could be the indiscriminate use of antibiotics in the area for treatment of mastitis providing selective pressure on the bacteria for acquisition of resistance. Poor sensitivity to Chloramphenicol and B-lactam group of antibiotics have been reported by others also [26, 27]. But to our knowledge, this is the first time in India that a high level (27.8%) of resistance to Azithromycin by *S*. *aureus* is reported. This is of concern since Azithromycin is a critical important drug in treatment of bacterial infections in humans. High level of resistance to erythromycin has been reported in S. aureus (33.1%) and CNS (67%) in China, probably as a result of extensive use of macrolide antibiotics in dairy cows of that region [28].

High prevalence of Staphylococcal infection in the udder of dairy cows have been reported in large dairy herds before (1) where chances of contagious transmission are high. But high prevalence rates (61.14%) in small holder systems in our study, which are isolated from each other and employ individual milkers indicate the Staphylococcal infection to be endemic among the entire cattle population of the region under study. This, coupled with the antibiotic resistance pattern and virulence of some of the isolates indicate a serious threat to human health in the area by infection with the Staphylococci as such, or by the potential transmissibility of specific resistant clones or antimicrobial resistance determinants to pathogens in humans. Environmental contamination by MRSA from veal calves was found to be a major source of infection to farmers and members of their household [3]. Genes for resistance to β-lactam group of antibiotics like methicillin are assumed to have originated in strains of *S*. *fleurettii* primarily found in animals, and have spread to human pathogens from that source [29]. PVL toxin, on the other hand, is reported to be having a major role in pathogenesis of CA-MRSA in human patients, but not in animal models [30].

Currently, staphylococci other than *S*. *aureus* comprise 39 characterized species. More than 10 CNS species have been isolated from mastitis, but only a few species predominate. The groEL chaperone has been identified as a heat shock protein essential for the survival of bacteria, and PCR-RFLP of groEL gene is a valuable tool for accurate identification of Staphylococci independent of origin of the isolate [31]. The PCR-RFLP patterns of *groEL* gene obtained in this study have close resemblance with the patterns obtained with typed strains of *S*. *chromogenes* and *S*. *hyicus*. Double digestion with HindIII and PvuII enzymes have been recommended for species level identification by PCR-RFLP of these two species [31]. Since we have used only PvuII for digestion, further confirmation may be required for the identity of these bacteria. Detection of *S*. *hyicus* in our study is of concern since this species has been occasionally associated with severe infections, but not all the strains have the same virulence characteristics [32].

In conclusion, dairy cattle in the area of study had high rates of infection by antibiotic resistant bacteria possessing virulent genes which may hold a serious threat to human health. Our findings point to the need for conjointly considering the molecular level identity and antimicrobial profile of Staphylococci in dairy cattle as an important aspect of epidemiological studies for therapeutic management and control in humans.

## Supporting information

S1 Fig Antimicrobial resistance to Oxacillin and Cefoxitin identified by strip method of Staphylococci isolated from dry cows in India. (DOCX)

S2 Fig Amplicons of the *groEL* gene by PCR of coagulase negative Staphylococci isolated from dry cows in India. (DOCX)

S3 Fig Restriction Fragment Length Ploymorphism patterns of *groEL* gene by PCR-RFLP of coagulase negative Staphylococci isolated from dry cows in India. (DOCX)

S4 Fig Amplicons of the *blaZ* gene in *Staphylococcus aureus*. (DOCX)

S5 Fig Amplicons of the *mecA* gene in *Staphylococcus aureus*. (DOCX)
