# Supplementary material for: Genotypic and phenotypic β-lactam resistance and presence of PVL gene in Staphylococci from dry bovine udder
Source: PLoS One. 2017 Nov 1;12(11):e0187277. doi: 10.1371/journal.pone.0187277 (PMC5665534; doi:10.1371/journal.pone.0187277)

S1 Fig. Antimicrobial resistance to Oxacillin and Cefoxitin identified by strip method of Staphylococci isolated from dry cows in India


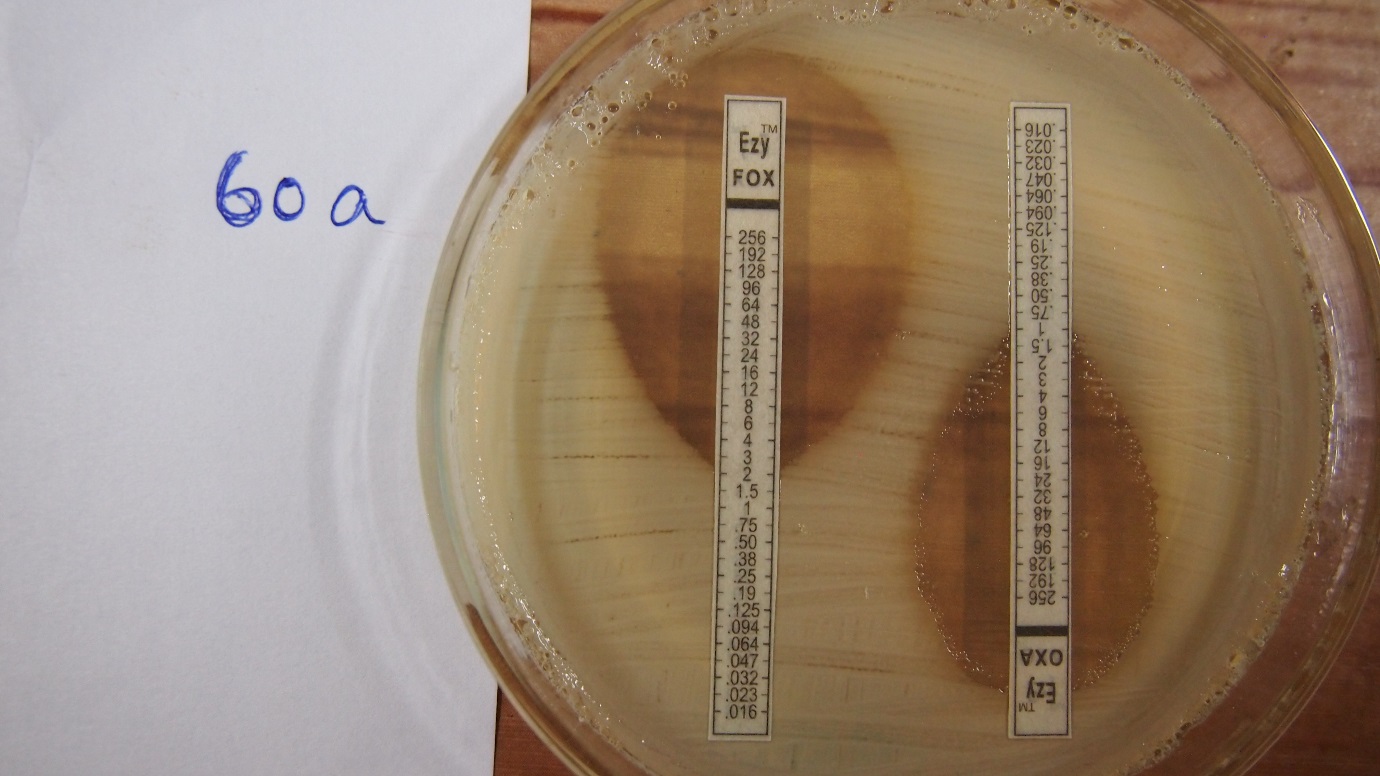

Supplement: S1 Fig — (DOCX) [file pone.0187277.s001.docx]
