# Supplementary material for: Genotypic and phenotypic β-lactam resistance and presence of PVL gene in Staphylococci from dry bovine udder
Source: PLoS One. 2017 Nov 1;12(11):e0187277. doi: 10.1371/journal.pone.0187277 (PMC5665534; doi:10.1371/journal.pone.0187277)

S2 Fig. Amplicons of the *groEL* gene by PCR of coagulase negative Staphylococci isolated from dry cows in India.

M=1kb plus ladder (Fermentas Germany)


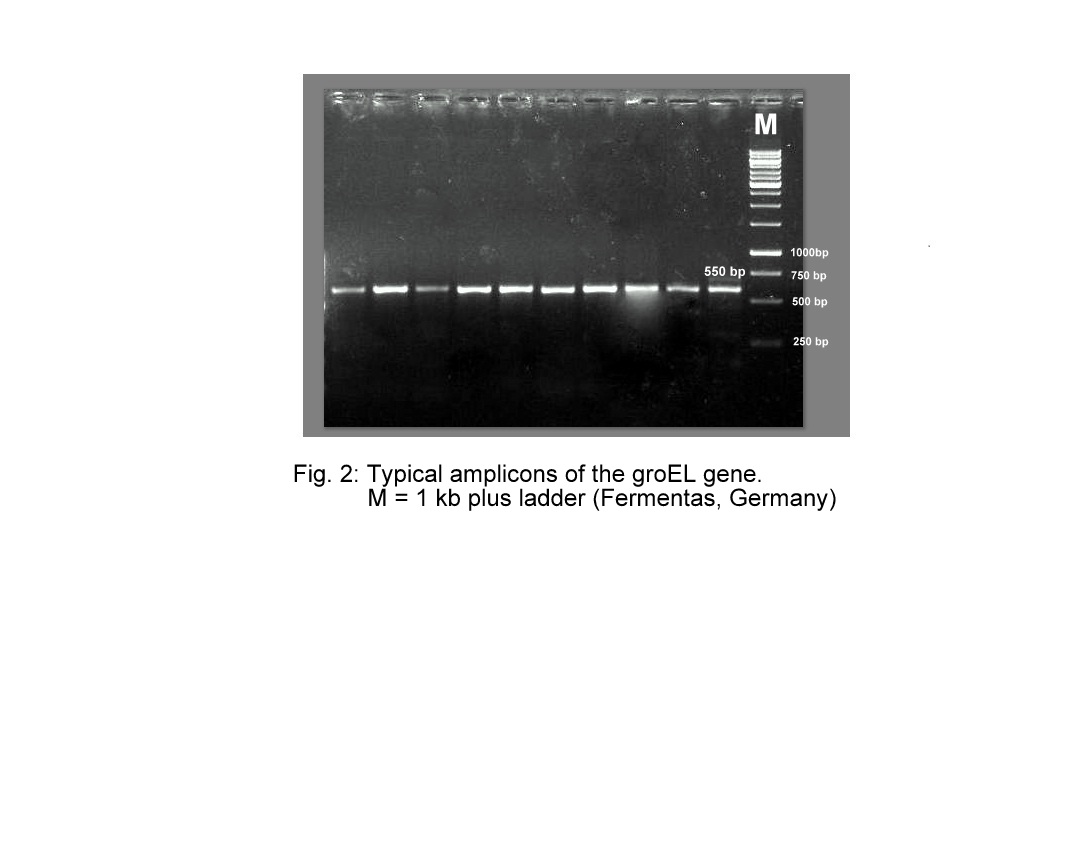

Supplement: S2 Fig — (DOCX) [file pone.0187277.s002.docx]
