# Supplementary material for: Genotypic and phenotypic β-lactam resistance and presence of PVL gene in Staphylococci from dry bovine udder
Source: PLoS One. 2017 Nov 1;12(11):e0187277. doi: 10.1371/journal.pone.0187277 (PMC5665534; doi:10.1371/journal.pone.0187277)

S3 Fig. Restriction Fragment Length Ploymorphism patterns of *groEL* gene by PCR-RFLP of coagulase negative Staphylococci isolated from dry cows in India.

Lane 1-5 and 7-11 are digested PCR products from CNS.

M=50bp ladder (Fermentas Germany)


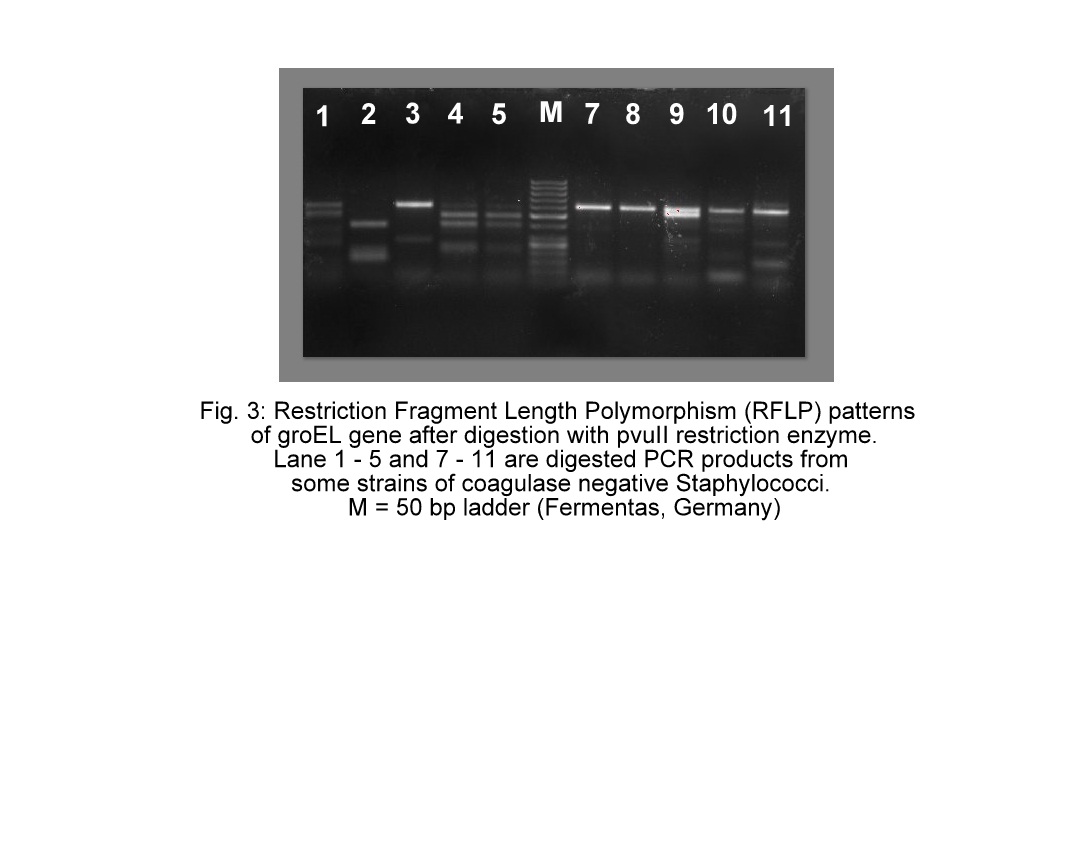

Supplement: S3 Fig — (DOCX) [file pone.0187277.s003.docx]
