# Supplementary figures and images for: Genotypic and phenotypic β-lactam resistance and presence of PVL gene in Staphylococci from dry bovine udder
Source: PLoS One. 2017 Nov 1;12(11):e0187277. doi: 10.1371/journal.pone.0187277 (PMC5665534; doi:10.1371/journal.pone.0187277)

S4 Fig. Amplicons of the *blaZ* gene in *Staphylococcus aureus*.

M= 100bp (SIGMA USA)


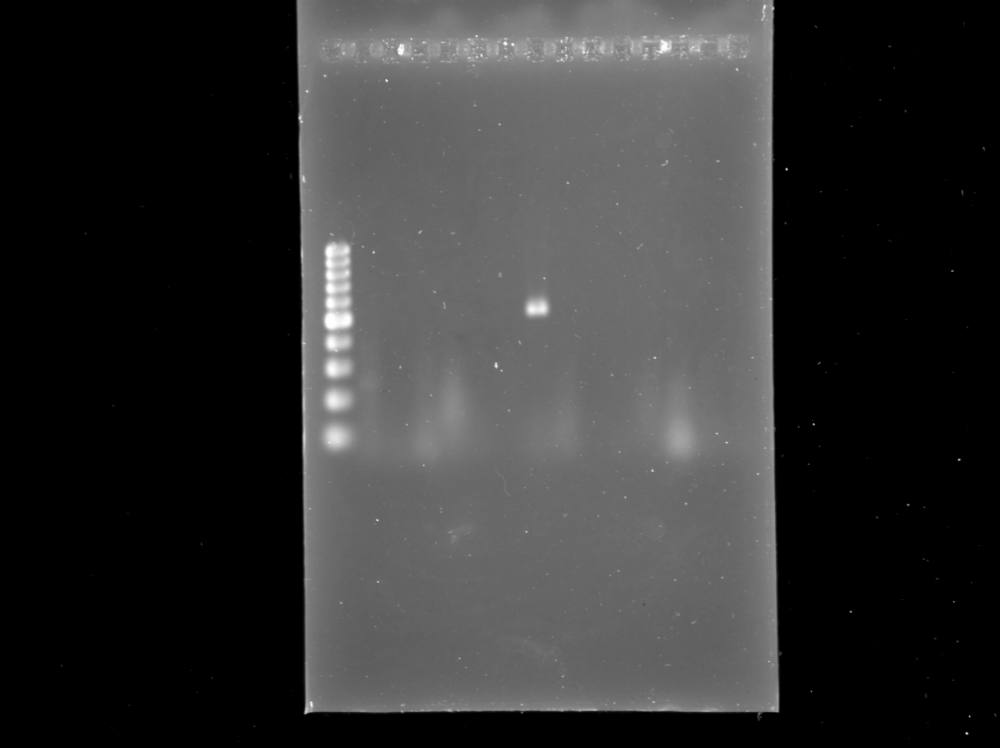

Supplement: S4 Fig — (DOCX) [file pone.0187277.s004.docx]

S5 Fig. Amplicons of the *mecA* gene in *Staphylococcus aureus*.

M=50bp (Sigma, USA)


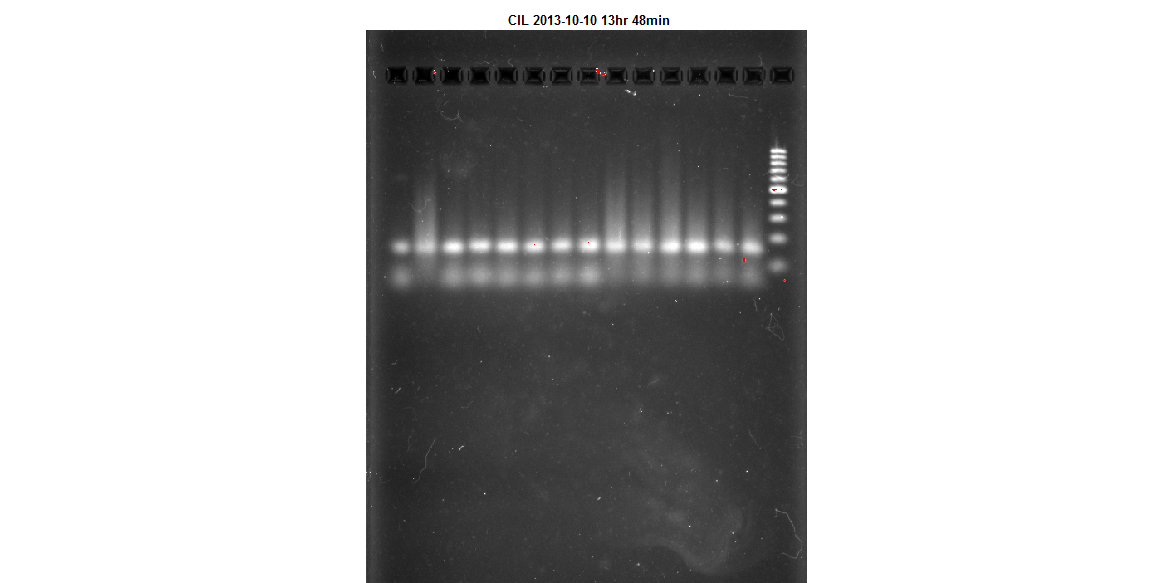

Supplement: S5 Fig — (DOCX) [file pone.0187277.s005.docx]
